# Cerebrospinal fluid TNF-α, regional degree centrality, and depressive symptoms after herpes simplex encephalitis: a cross-sectional observational study

**DOI:** 10.3389/fneur.2026.1942369

**Published:** 2026-10-16

**Authors:** Jingyang Ma, Yuying Li, Yumei Zhang, Yangli Yu, Ying Bai, Yuanfei Chen, Yayun Liao, Chunyan Zheng, Jueyue Yan, Qinglong Feng, Chengze Chen

**Affiliations:** 1 Organ Donation Coordination Office, Shulan (Hangzhou) Hospital, Shulan International Medical College, Hangzhou, Zhejiang, China; 2 Department of Medicine, Quzhou College of Technology, Quzhou, China; 3 Intensive Care Unit, Quzhou Kecheng People's Hospital, Quzhou, Zhejiang, China; 4 Department of Critical Care Medicine, The First Affiliated Hospital, Zhejiang University School of Medicine, Hangzhou, Zhejiang, China; 5 Central Health Center of Nianli Town, Quzhou, Zhejiang, China; 6 Quzhou Second People's Hospital, Quzhou, Zhejiang, China

**Keywords:** cerebrospinal fluid, degree centrality, depression, herpes simplex encephalitis, resting-state functional MRI, tumor necrosis factor-α

## Abstract

**Background:**

Herpes simplex encephalitis (HSE) survivors frequently experience neuropsychiatric sequelae, including depressive symptoms, but the biological correlates underlying post-HSE depression remain incompletely understood. Neuroinflammation and altered functional brain organization have been implicated in depression; however, their relationships after HSE have not been characterized. This study investigated the associations among cerebrospinal fluid (CSF) tumor necrosis factor-α (TNF-α), resting-state functional brain connectivity, and depressive symptoms in HSE survivors.

**Methods:**

This two-center cross-sectional observational study included adults with HSE and healthy controls recruited between June 2023 and June 2025. Participants were classified into healthy controls (HC), HSE without depression (HSE-ND), and HSE with depression (HSE-D) groups. Depressive symptoms were assessed using the 17-item Hamilton Depression Rating Scale (HAMD-17). CSF inflammatory cytokines were measured, and resting-state functional MRI was performed. CSF TNF-α was prespecified as the primary cytokine for the imaging, hierarchical regression, and exploratory mediation analyses; the other cytokines were evaluated in secondary group-comparison and symptom-correlation analyses. Voxel-wise degree centrality (DC) analysis was used to identify functional network alterations associated with post-HSE depression. Associations among CSF TNF-α, regional DC values, and depressive symptom severity were further evaluated using correlation, regression, and exploratory mediation analyses.

**Results:**

A total of 74 participants were included (24 HC, 27 HSE-ND, and 23 HSE-D). CSF TNF-α concentrations were higher in HSE-D than in HC and HSE-ND and were positively associated with HAMD-17 scores in the HSE cohort. The primary whole-brain voxel-wise contrast identified candidate clusters with lower DC in the right amygdala, right insula, left fusiform gyrus, and left postcentral gyrus. In secondary regional comparisons, HSE-D versus HSE-ND differences remained significant in the right amygdala and left postcentral gyrus but not in the left fusiform gyrus or right insula. CSF TNF-α was inversely associated with DC in the right amygdala, right insula, and left postcentral gyrus. Regional DC values were negatively correlated with depressive symptom severity. Exploratory hierarchical models showed greater in-sample explained variance after adding CSF TNF-α and regional DC to clinical covariates, but Model 3 was not a validated independent-predictor model. Standardized indirect-effect point estimates were 0.149 for the right amygdala, 0.081 for the left fusiform gyrus, 0.163 for the right insula, and 0.091 for the left postcentral gyrus. These exploratory estimates require validation.

**Conclusion:**

In HSE survivors, elevated CSF TNF-α and altered functional brain network organization were associated with depressive symptoms. The regional pattern is exploratory and should not be interpreted as specific to a primary depressive disorder, independent of neurological sequelae, or causal. Longitudinal studies are needed to determine temporal relationships, compartmental inflammatory correlates, and clinical relevance.

## Introduction

Herpes simplex encephalitis (HSE) is a severe central nervous system infection that causes substantial neurological morbidity. Although prompt antiviral therapy has improved survival, recovery is often incomplete. Survivors may experience persistent cognitive deficits, fatigue, mood symptoms, impaired social functioning, and reduced quality of life (1). Long-term memory and other cognitive impairments are particularly well documented after HSE (2). Depressive symptoms may emerge or persist during post-acute recovery and further complicate rehabilitation. Recognition is difficult because affective symptoms often coexist with cognitive deficits, anxiety, medication exposure, and residual neurological disability. Follow-up has traditionally emphasized survival, seizures, and cognitive outcomes, with less systematic attention to affective recovery. Conventional measures of acute disease severity and structural injury do not fully explain the neuropsychiatric outcomes observed after HSE. Consequently, the mechanisms and clinical correlates of post-HSE depression remain poorly understood.

Neuroinflammatory abnormalities have been linked to behavioral and affective symptoms across infectious, inflammatory, and acquired neurological disorders. CSF studies in major depressive disorder also support an association between central inflammatory markers, including TNF-α, and depression (3). TNF-α is a key proinflammatory cytokine in systemic and central immune responses. CSF TNF-α may reflect inflammatory signaling within the central nervous system, but it cannot identify the cellular source or demonstrate active parenchymal inflammation. Its clinical meaning may vary with disease stage, treatment exposure, neurovascular integrity, and residual tissue injury. Prior HSE studies have largely examined acute CSF cytokine responses, virological findings, and short-term neurological outcomes (4). Whether CSF TNF-α is associated with depressive symptoms during post-acute recovery remains uncertain.

Neuroimaging studies of HSE have focused mainly on structural lesions, regional atrophy, and cognitive impairment. These approaches characterize focal injury but may not capture disruption of distributed functional organization. Resting-state fMRI measures spontaneous correlations in blood oxygen level-dependent signals without requiring task performance. Degree centrality (DC) is a voxel-wise graph measure of each voxel’s connectivity with the rest of the brain (5). It can identify altered network centrality without an *a priori* seed. DC abnormalities have been reported in depression, including reduced insular centrality (6), but their relevance to post-HSE depression is unknown. Integrating CSF TNF-α with functional imaging may show whether variation in this central-compartment marker accompanies altered brain network organization. The relationship among CSF TNF-α, functional network changes, and depressive symptoms after HSE has not been adequately characterized.

This observational study compared depressive symptoms, CSF concentrations of TNF-α, IL-6, IL-1β, IL-10, and IL-8, and resting-state functional connectivity among HSE-D, HSE-ND, and healthy controls. Before analysis, CSF TNF-α was prespecified as the primary inflammatory variable for the imaging, hierarchical regression, and exploratory mediation analyses. The remaining cytokines were retained for secondary group comparisons and symptom-correlation analyses. We first characterized the clinical and CSF cytokine features associated with post-HSE depression. We then examined associations of CSF TNF-α with voxel-wise and regional DC and assessed relationships between regional DC and symptom severity. Comparing the HSE groups identified features associated with depression against a shared history of encephalitis. Healthy controls provided reference values for regional DC and CSF cytokine concentrations. Hierarchical regression evaluated the incremental associations of clinical variables, CSF TNF-α, and regional DC with HAMD-17 scores. Exploratory mediation analyses estimated statistical indirect effects involving CSF TNF-α, regional DC, and depressive symptoms. These analyses characterized associations and were not interpreted as causal.

## Methods

### Study design and participants

This two-center cross-sectional observational study included adults with HSE and healthy controls (HC) recruited between June 2023 and June 2025 at the First Affiliated Hospital, Zhejiang University School of Medicine, and Quzhou Kecheng People’s Hospital. HSE was diagnosed according to International Encephalitis Consortium criteria (7) and confirmed by polymerase chain reaction detection of herpes simplex virus DNA in CSF. Patients were assessed during a clinically stable, post-acute follow-up phase. Exclusion criteria included a major pre-existing neurological or psychiatric disorder, an unrelated structural brain disorder, an MRI contraindication, or incomplete clinical or neuropsychological assessment. Participants were also excluded for incomplete MRI data or unacceptable image quality. HC participants had no major neurological or psychiatric disorder, active inflammatory disease, or clinically relevant structural MRI abnormality.

Participants were classified as HC, HSE without depression (HSE-ND), or HSE with depression (HSE-D). Trained clinicians diagnosed depressive disorders through structured interviews based on DSM-5 criteria. The 17-item Hamilton Depression Rating Scale (HAMD-17) measured symptom severity (8) and was not the sole criterion for group assignment. The institutional ethics committee approved the protocol. All participants or their legally authorized representatives provided written informed consent. The specific DSM-5 depressive-disorder subtype was recorded from the structured clinical assessment where available; HAMD-17 quantified current symptom severity and did not define the diagnostic category.

### Clinical assessment

Recorded demographic and clinical variables included age, sex, education, body mass index, disease duration, hospitalization duration, acute treatment, and intensive care unit admission. We also recorded mechanical ventilation, medication exposure at MRI, and postdischarge rehabilitation. Disease duration was defined as the interval from symptom onset to study assessment. The Hamilton Anxiety Rating Scale (HAMA) measured anxiety symptoms (9). Global cognition was assessed with the Mini-Mental State Examination (MMSE) (10) and Montreal Cognitive Assessment (MoCA) (11). Trained personnel completed the clinical scales, CSF sampling, and MRI within the same assessment period.

### Laboratory measurements

CSF was obtained by lumbar puncture during the assessment visit. Samples were centrifuged, aliquoted, and stored at −80 °C until analysis. CSF TNF-α concentrations were measured in duplicate using a sandwich enzyme-linked immunosorbent assay.

CSF IL-6, IL-1β, IL-10, and IL-8 concentrations were quantified using a multiplex bead-based immunoassay. Samples from all three groups were processed using identical procedures. Only CSF inflammatory biomarkers were analyzed; no peripheral blood, plasma, serum, or hs-CRP measurements were included in the present study.

### MRI acquisition

MRI was performed on a 3.0-T GE Signa HDxt system (GE Healthcare) with an 8-channel phased-array head coil. Foam padding minimized head motion. During resting-state acquisition, participants kept their eyes closed, remained awake, relaxed, and avoided structured thinking. Wakefulness was confirmed after the scan.

Resting-state blood oxygen level-dependent images were acquired using gradient-echo echo-planar imaging. Parameters were repetition time, 2,000 ms; echo time, 30 ms; flip angle, 90°; field of view, 240 × 240 mm^2^; and matrix, 64 × 64. The acquisition included 37 interleaved ascending axial slices, 4.0-mm thickness, no gap, and 3.75 × 3.75 × 4.0 mm^3^ voxels. Phase encoding was anterior to posterior, and 210 volumes were acquired over 7 min.

Three-dimensional sagittal T1-weighted images were acquired with an inversion-recovery-prepared spoiled gradient-echo sequence (BRAVO). Parameters were repetition time, 8.2 ms; echo time, 3.2 ms; inversion time, 450 ms; flip angle, 12°; field of view, 256 × 256 mm^2^; and matrix, 256 × 256. The acquisition included 188 contiguous slices, 1.0-mm isotropic voxels, and an ASSET factor of 2. Axial T2-weighted FLAIR images used repetition time, 9,000 ms; echo time, 120 ms; inversion time, 2,500 ms; and refocusing angle, 160°. Additional parameters were a 240 × 240 mm^2^ field of view, 256 × 256 matrix, 36 slices, 4.0-mm thickness, 0.5-mm gap, and 0.94 × 0.94 × 4.0 mm^3^ voxels (Supplementary Table S1). HSE-related FLAIR lesions were delineated manually or semiautomatically and reviewed by a neuroradiologist. Total lesion volume was used in sensitivity analyses.

### MRI preprocessing and degree centrality analysis

Resting-state fMRI data were preprocessed in MATLAB with Statistical Parametric Mapping version 12 and the Data Processing and Analysis for Brain Imaging toolbox (12). The first 10 volumes were discarded, leaving 200 volumes. Remaining images underwent slice-timing correction, rigid-body realignment, and co-registration of the T1-weighted image to the mean functional image. Tissue segmentation and DARTEL normalization to Montreal Neurological Institute space were then performed (13). Images were resampled to 3 × 3 × 3 mm^3^. Participants were excluded if maximum translation exceeded 2.0 mm, rotation exceeded 2.0°, or more than 20% of volumes had framewise displacement above 0.5 mm. Mean framewise displacement and censored-volume count were included in voxel-wise models to reduce motion-related bias (14).

Nuisance regression included the Friston 24-parameter motion model and mean white-matter and CSF signals. Global signal regression was not performed. Images were smoothed with a 6-mm full-width at half-maximum Gaussian kernel, linearly detrended, and temporally filtered at 0.01–0.08 Hz.

Voxel-wise DC was calculated within a whole-brain gray-matter mask according to established centrality methods (5). Pearson correlations were computed between the residual time series of every voxel pair. In the primary binary analysis, correlations greater than *r* = 0.25 were retained. Negative correlations and values at or below this threshold were set to zero. Each voxel’s DC equaled the number of retained positive connections with all other gray-matter voxels. Individual maps were standardized within the mask by subtracting the spatial mean and dividing by the spatial standard deviation. This procedure produced z-standardized DC maps (zDC).

The primary whole-brain contrast compared HSE-D with HSE-ND using a voxel-wise general linear model. Covariates were age, sex, education, mean framewise displacement, and censored-volume count. Multiple comparisons were controlled with Gaussian random field correction. The two-sided voxel-defining threshold was *p* < 0.001, and the cluster-level corrected threshold was *p* < 0.05. Mean zDC values were extracted from corrected clusters in the right amygdala, left fusiform gyrus, right insula, and left postcentral gyrus. Regional values were compared across HC, HSE-ND, and HSE-D using one-way analysis of variance and Holm-adjusted pairwise comparisons. Because the same cohort defined these regions, subsequent regional analyses were considered exploratory.

### Statistical analysis

Analyses were performed in R version 4.4.3. Continuous variables are presented as mean ± standard deviation or median (interquartile range); categorical variables are presented as number (percentage). Three-group comparisons used one-way analysis of variance or Kruskal-Wallis tests for continuous variables. Categorical variables were compared with *χ*^2^ or Fisher exact tests. Significant omnibus tests were followed by Holm-adjusted pairwise tests or Dunn tests, as appropriate.

Partial correlations between CSF TNF-α and regional zDC were adjusted for age, sex, education, and log-transformed disease duration. Correlations between regional zDC and HAMD-17 were adjusted for CSF TNF-α. As an additional sensitivity analysis addressing possible anxiety confounding, HAMA score was added to the relevant HAMD-17 association models. A separate matrix containing CSF TNF-α, HAMD-17, MMSE, and four regional zDC measures included 21 pairwise tests.

Hierarchical linear regression models used HAMD-17 score as the dependent variable. Model 1 included age, sex, education, MMSE score, and log-transformed disease duration. Model 2 added log-transformed CSF TNF-α. Model 3 added right amygdala and right insula zDC. Given the sample size and the same-sample derivation of the imaging regions, Model 3 was treated as exploratory rather than as a confirmatory predictor model. We report coefficients, 95% confidence intervals, *p* values, *R*^2^, adjusted *R*^2^, and changes in *R*^2^.

Exploratory single-region mediation models specified log-transformed CSF TNF-α as the exposure, regional zDC as the intermediate variable, and HAMD-17 score as the outcome. Indirect effects were estimated with 5,000 bias-corrected and accelerated bootstrap resamples. CSF TNF-α was the prespecified primary cytokine for this analysis. Robustness analyses varied resampling and interval specifications and added clinical and imaging covariates. Further analyses used medication-related exclusions, CSF TNF-α winsorization, parallel-region models, reverse-direction models, and an MMSE negative-control outcome. All tests were two-sided, with a nominal threshold of *p* < 0.05 unless multiplicity adjustment was specified. Primary analyses used complete cases.

## Results

### Participant characteristics

Between June 2023 and June 2025, 103 participants were assessed for eligibility from the First Affiliated Hospital, Zhejiang University School of Medicine, and Quzhou Kecheng People’s Hospital. Of these, 38 were healthy controls and 65 were patients with HSE. After excluding 14 controls and 15 patients with HSE, 24 healthy controls and 50 patients with HSE were included in the final analysis, consisting of 27 HSE patients without depression (HSE-ND) and 23 HSE patients with depression (HSE-D) (Figure 1; Table 1).

**Figure 1:**
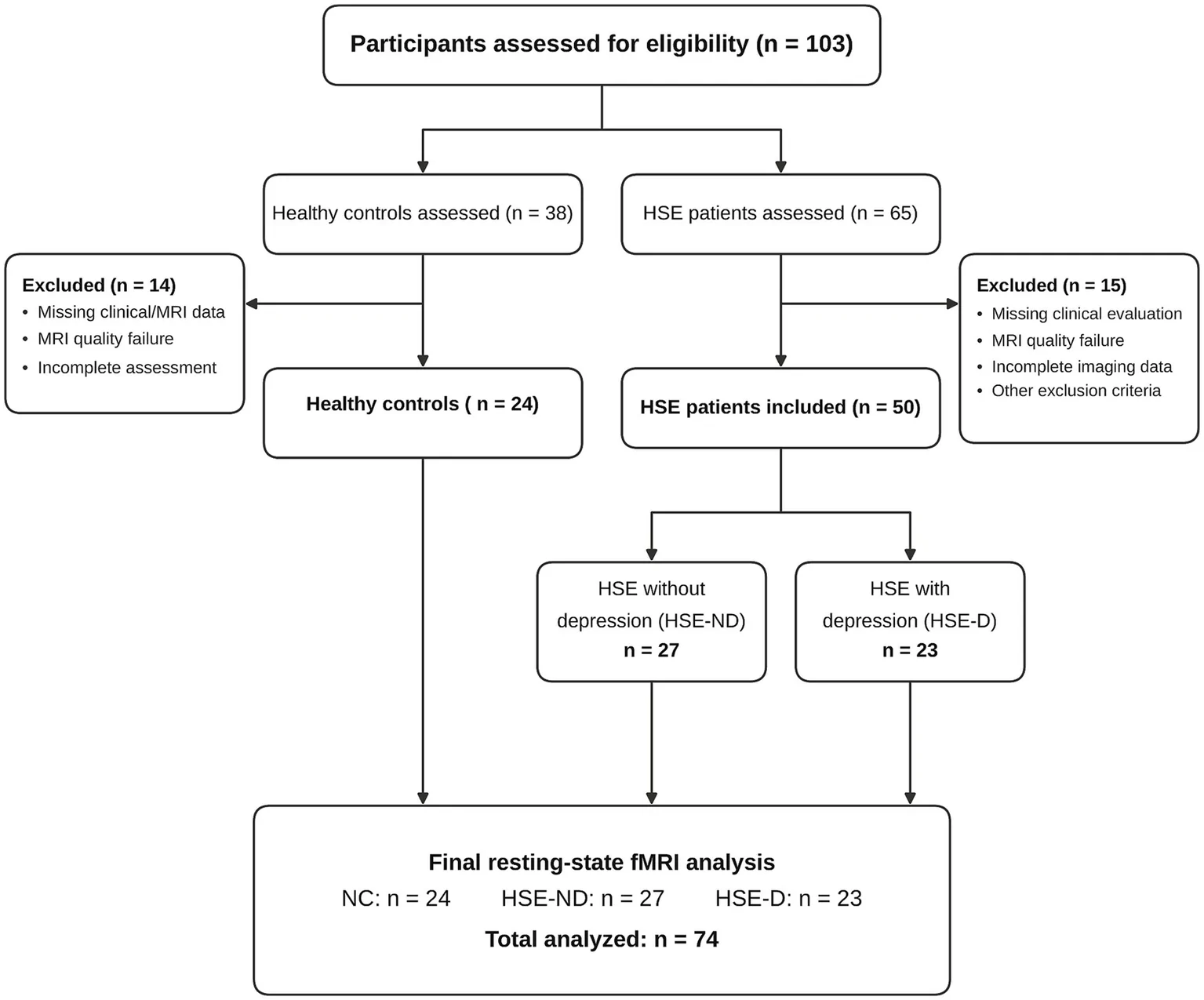
Participant recruitment and study inclusion. Flow diagram showing participant selection between June 2023 and June 2025. The final analysis included 24 healthy controls (HC), 27 patients with herpes simplex encephalitis without depression (HSE-ND), and 23 patients with HSE with depression (HSE-D).

**Table 1:** Demographic and clinical characteristics of study participants.

Characteristic	Healthy controls (*n* = 24)	HSE without depression (HSE-ND, *n* = 27)	HSE with depression (HSE-D, *n* = 23)	*p* value
Number of participants	24	27	23	–
Age, years	50.0 ± 14.8	48.1 ± 13.1	47.7 ± 12.4	0.815
Sex, male, *n* (%)	13 (54.2)	12 (44.4)	10 (43.5)	0.713
Education, years	13.0 ± 2.9	12.4 ± 3.8	13.3 ± 2.9	0.588
BMI, kg/m^2^	24.0 ± 3.5	23.3 ± 2.9	24.1 ± 3.2	0.638
Disease duration, months	–	13.7 (9.5–19.9)	15.3 (9.9–24.2)	0.448
Hospitalization duration, days	–	18.5 (13.6–23.2)	23.9 (18.5–26.8)	0.041
MMSE score	27.1 ± 2.1	26.6 ± 2.3	24.8 ± 2.7b	0.009
HAMD-17 score	5.5 (3.2–7.9)	6.5 (3.2–11.9)	12.8 (8.1–15.6)	0.002
HAMA score	5.1 (1.1–10.4)	6.4 (3.4–10.4)	11.5 (8.0–14.4)	0.005
MoCA score	26.7 ± 2.4	25.5 ± 2.5	24.0 ± 3.3b	0.019
CSF TNF-α, pg/mL	7.8 (6.4–10.4)	10.5 (6.7–13.0)	14.9 (8.8–20.0)	0.012
Antiviral treatment, *n* (%)	–	27 (100.0)	23 (100.0)	–
Corticosteroid treatment, *n* (%)	–	8 (29.6)	9 (39.1)	0.557

Age, sex, education, and body mass index did not differ among groups (all *p* > 0.05). Median hospitalization was longer in HSE-D than in HSE-ND (23.9 [IQR, 18.5–26.8] vs. 18.5 [IQR, 13.6–23.2] days; *p* = 0.041). Disease duration was similar between the HSE groups (*p* = 0.448). MMSE scores differed among HC, HSE-ND, and HSE-D (27.1 ± 2.1, 26.6 ± 2.3, and 24.8 ± 2.7; *p* = 0.009). Corresponding HAMD-17 scores were 5.5 [IQR, 3.2–7.9], 6.5 [IQR, 3.2–11.9], and 12.8 [IQR, 8.1–15.6] (*p* = 0.002). HAMA scores were 5.1 [IQR, 1.1–10.4], 6.4 [IQR, 3.4–10.4], and 11.5 [IQR, 8.0–14.4] (*p* = 0.005). MoCA scores were 26.7 ± 2.4, 25.5 ± 2.5, and 24.0 ± 3.3, respectively (*p* = 0.019). *Post hoc* comparisons showed lower MMSE and higher HAMD-17 and HAMA scores in HSE-D than in both comparison groups. MoCA was lower in HSE-D than in HC. All HSE participants received antiviral treatment. Corticosteroid use did not differ between HSE-D and HSE-ND (39.1% vs. 29.6%; *p* = 0.557) (Table 1). Other acute treatments, intensive care admission, mechanical ventilation, antiseizure medication, and rehabilitation did not differ between the HSE groups. Current antidepressant use was more frequent in HSE-D (26.1% vs. 3.7%; *p* = 0.039) (Supplementary Table S2). DSM-5 subtype counts were major depressive disorder, *n* = 3; depressive disorder due to another medical condition, *n* = 3; adjustment disorder with depressed mood, *n* = 4; and other or unspecified depressive disorder, *n* = 13. HAMD-17 was analyzed as a continuous measure of current symptom severity and was not used as the sole diagnostic criterion; the median score of 12.8 (IQR, 8.1–15.6) describes symptom burden rather than diagnostic classification.

### CSF cytokine concentrations

CSF TNF-α concentrations differed among HSE-D, HSE-ND, and HC groups (*p* = 0.012). Median concentrations were 14.9 [IQR, 8.8–20.0], 10.5 [IQR, 6.7–13.0], and 7.8 [IQR, 6.4–10.4] pg/mL, respectively. Concentrations were higher in HSE-D than in HC (*p* = 0.010) and HSE-ND (*p* = 0.043). CSF IL-6 concentrations also differed among groups (*p* = 0.021), with higher concentrations in HSE-D than in HC (*p* = 0.018). CSF IL-1β, IL-10, and IL-8 concentrations showed no overall group differences (all *p* > 0.05) (Figure 2; Supplementary Table S3).

**Figure 2:**
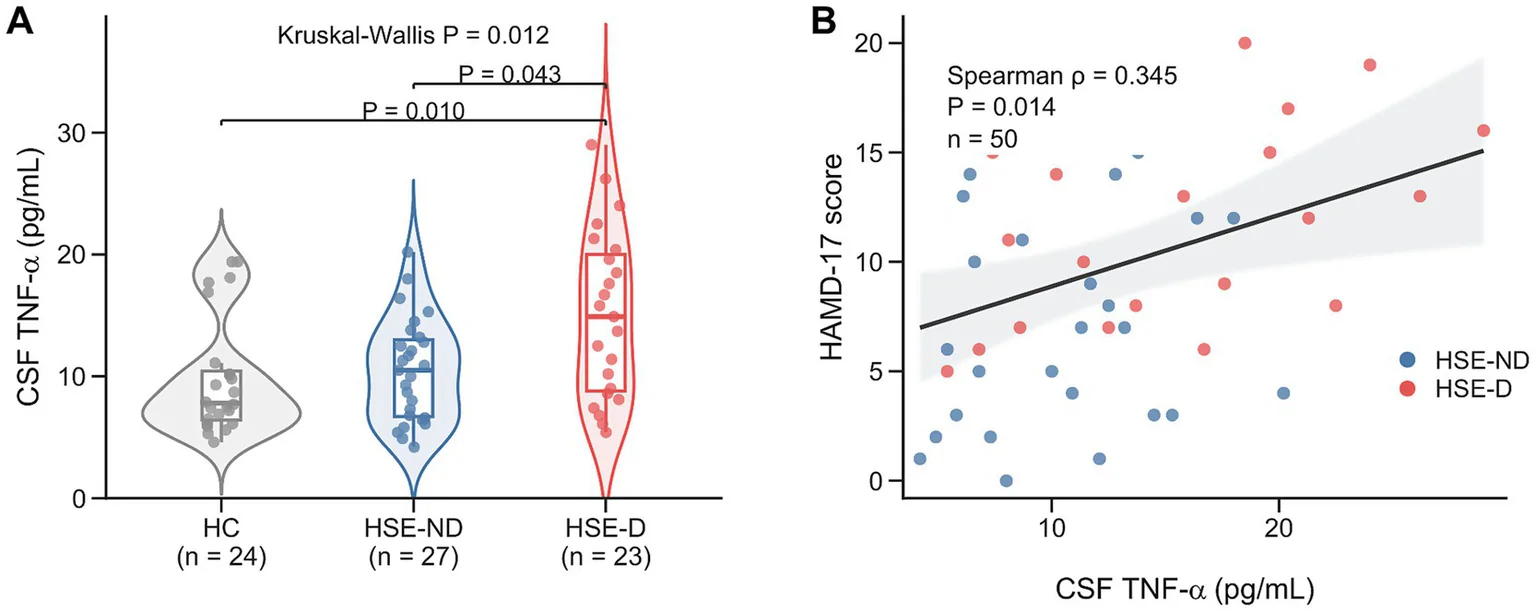
Cerebrospinal fluid TNF-α levels and depressive symptom severity. **(A)** CSF TNF-α concentrations among HC, HSE-ND, and HSE-D groups. **(B)** Correlation between CSF TNF-α concentration and HAMD-17 scores in patients with HSE. Violin plots show data distributions, with individual values displayed.

### Functional brain network alterations

The primary HSE-D versus HSE-ND contrast identified four clusters with lower DC after cluster-level Gaussian random field correction. Lower DC was observed in the right amygdala (91 voxels; MNI coordinates, 24, −3, −18; peak T = −4.21; corrected *p* = 0.018). The right insula cluster contained 86 voxels (36, 15, 0; peak T = −4.05; *p* = 0.024). The left fusiform cluster contained 73 voxels (−39, −48, −18; peak T = −3.86; *p* = 0.031). The left postcentral cluster contained 69 voxels (−42, −24, 51; peak T = −3.68; *p* = 0.044). Candidate clusters in the left hippocampus, right parahippocampal gyrus, left superior temporal gyrus, right precuneus, and left medial superior frontal gyrus did not survive correction. Figure 3 displays the secondary three-group ANOVA/F-statistic characterization, whereas the primary binary T-contrast cluster statistics are reported here and in Supplementary Table S4. Regional zDC differed among groups in all four corrected clusters. Group differences were observed in the right amygdala (F[2, 71] = 5.28; *p* = 0.007) and left fusiform gyrus (F[2, 71] = 4.06; *p* = 0.021). Differences were also observed in the right insula (F[2, 71] = 3.92; *p* = 0.024) and left postcentral gyrus (F[2, 71] = 5.46; *p* = 0.006). In Holm-adjusted comparisons, HSE-D had lower zDC than HC in the right amygdala (*p* = 0.009) and left fusiform gyrus (*p* = 0.023). HSE-D also had lower zDC than HC in the right insula (*p* = 0.026) and left postcentral gyrus (*p* = 0.007). Compared with HSE-ND, HSE-D had lower zDC in the right amygdala (*p* = 0.042) and left postcentral gyrus (*p* = 0.046), whereas the fusiform (*p* = 0.104) and insula (*p* = 0.101) comparisons were not significant. No HC versus HSE-ND comparison remained significant (all *p* ≥ 0.353) (Supplementary Figure S1). Accordingly, the fusiform and insula findings are treated as exploratory candidate regions rather than depression-specific regional abnormalities.

**Figure 3:**
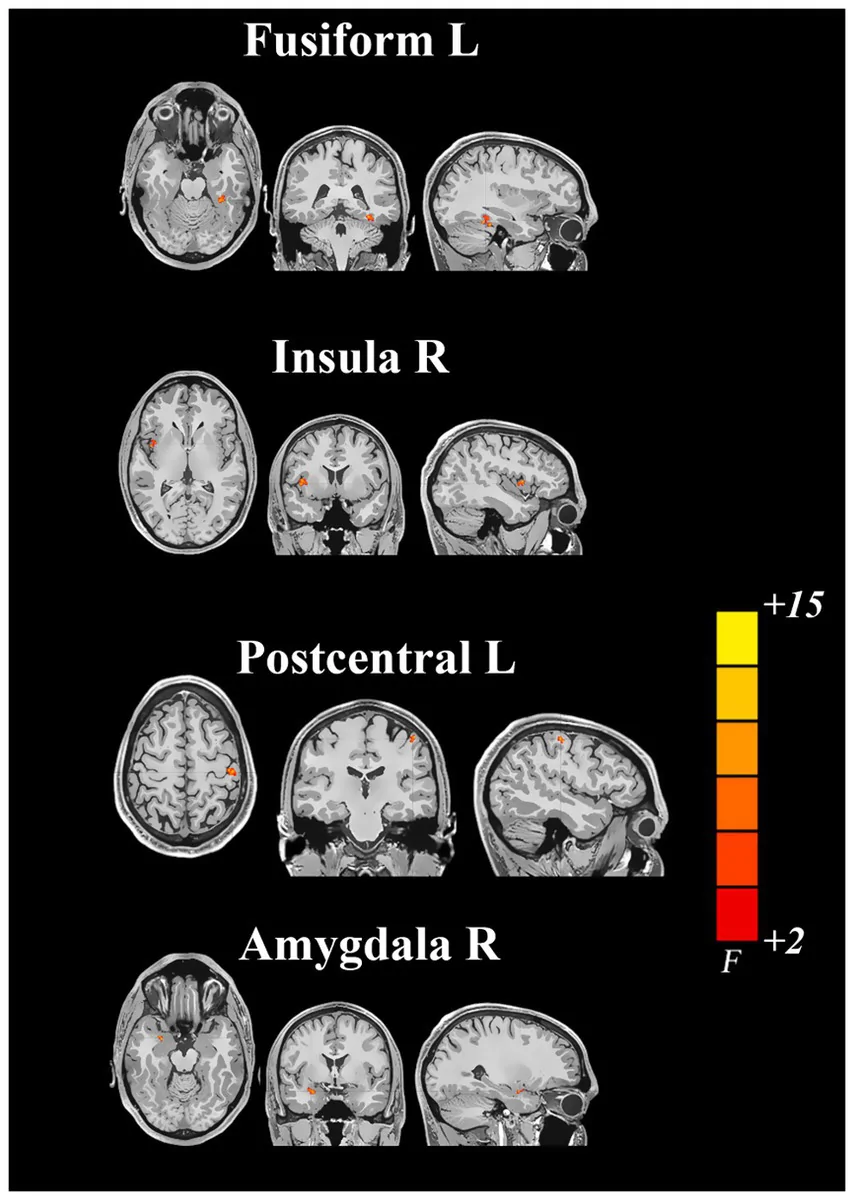
Brain regions showing altered degree centrality in HSE patients with depression. The displayed whole-brain map is the secondary three-group ANOVA/F-statistic characterization among HC, HSE-ND and HSE-D. The primary voxel-wise HSE-D versus HSE-ND T-contrast and its cluster-level GRF results are reported in the Results and Supplementary Table S4. Regional group distributions are shown in Supplementary Figure S1.

### Associations among CSF TNF-α, regional degree centrality, and depressive symptoms

In the pooled HSE cohort, CSF TNF-α correlated positively with HAMD-17 (Spearman rho = 0.345; *p* = 0.014). CSF IL-6 also correlated positively with HAMD-17 (rho = 0.288; *p* = 0.042). Because TNF-α was prespecified before analysis as the primary cytokine for imaging, regression, and exploratory mediation, only TNF-α was advanced to those analyses; IL-6 and the other cytokines remained secondary analyses. CSF IL-1β, IL-10, and IL-8 were not correlated with HAMD-17. No measured CSF cytokine correlated significantly with MMSE score, disease duration, or hospitalization duration (all *p* > 0.05) (Figure 2; Supplementary Table S6).

CSF TNF-α was inversely associated with zDC in the right amygdala (partial *r* = −0.355; *p* = 0.016), right insula (partial *r* = −0.411; *p* = 0.005), and left postcentral gyrus (partial *r* = −0.299; *p* = 0.044), but not in the left fusiform gyrus (partial *r* = −0.247; *p* = 0.098) (Figure 5). After adjustment for CSF TNF-α, HAMD-17 was inversely associated with right amygdala zDC (partial *r* = −0.421; *p* = 0.004), left fusiform zDC (partial *r* = −0.326; *p* = 0.027), right insula zDC (partial *r* = −0.397; *p* = 0.006), and left postcentral zDC (partial *r* = −0.305; *p* = 0.039) (Figure 4). Because HAMA scores differed between HSE-D and HSE-ND groups, we performed an additional sensitivity analysis by incorporating HAMA scores into the HAMD-17–zDC association models to evaluate the potential influence of anxiety symptoms. After adjustment for anxiety symptoms, the associations between HAMD-17 scores and regional zDC values remained directionally consistent, although the effect sizes were attenuated. Specifically, HAMD-17 scores remained inversely associated with right amygdala zDC (partial *r* = −0.172, *p* = 0.011), right insula zDC (partial *r* = −0.242, *p* = 0.036), left fusiform gyrus zDC (partial *r* = −0.129, *p* = 0.041), and left postcentral gyrus zDC (partial *r* = −0.217, *p* = 0.044). Furthermore, the positive association between CSF TNF-α and HAMD-17 scores remained significant after adjustment for HAMA scores (Spearman ρ = 0.272, *p* = 0.032). These findings suggest that anxiety symptoms may partially contribute to the observed associations but are unlikely to fully explain the relationships among CSF TNF-α, regional DC alterations, and depressive symptom severity. False discovery rate correction using the Benjamini–Hochberg procedure (15) was applied to the prespecified correlation matrix.

**Figure 4:**
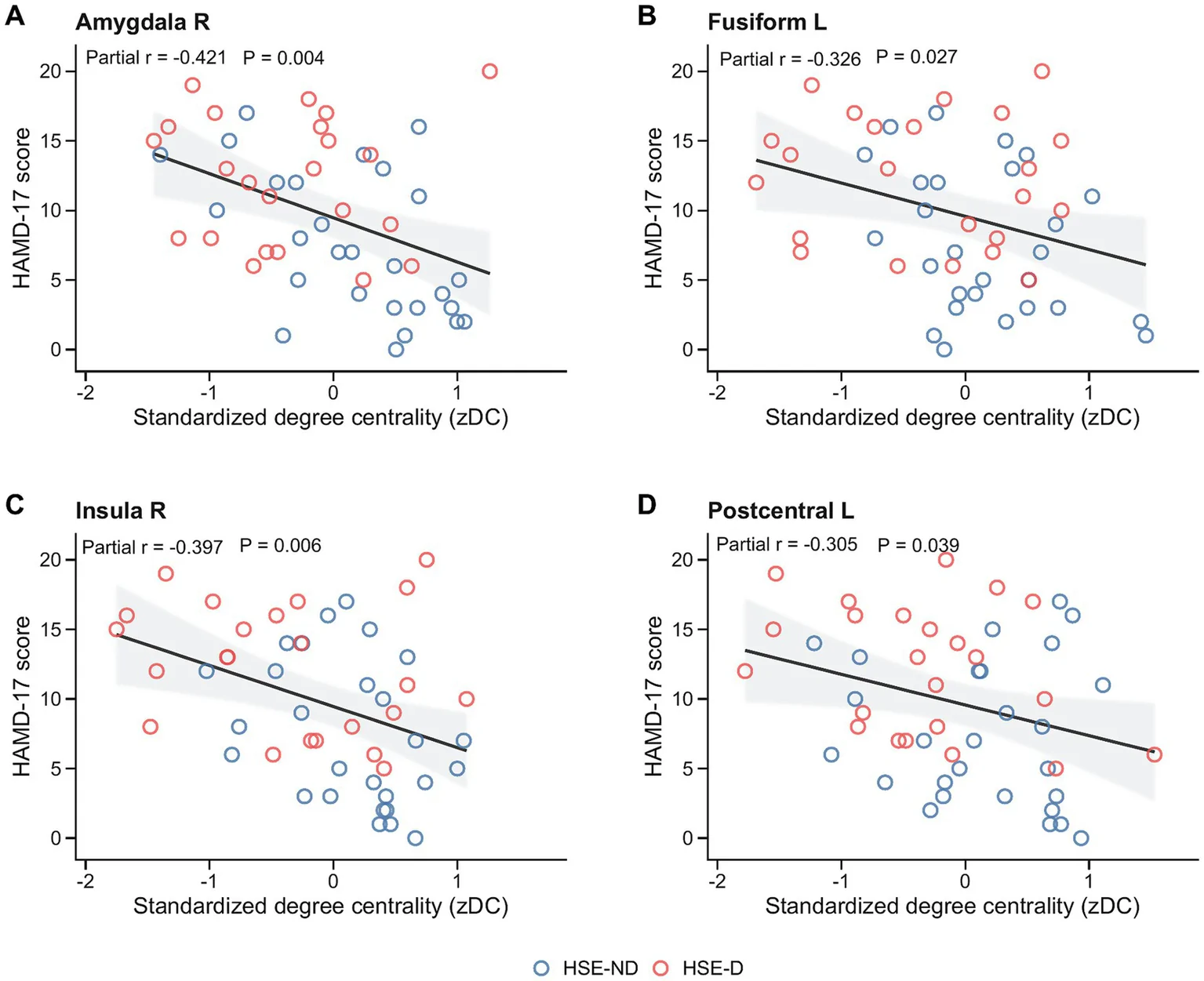
Associations between regional degree centrality and depressive symptoms. Partial correlation analyses between regional standardized degree centrality (zDC) and HAMD-17 scores in patients with HSE after adjustment for CSF TNF-α concentration. Each point represents one participant (*n* = 50). Regions included the right amygdala, left fusiform gyrus, right insula, and left postcentral gyrus.

**Figure 5:**
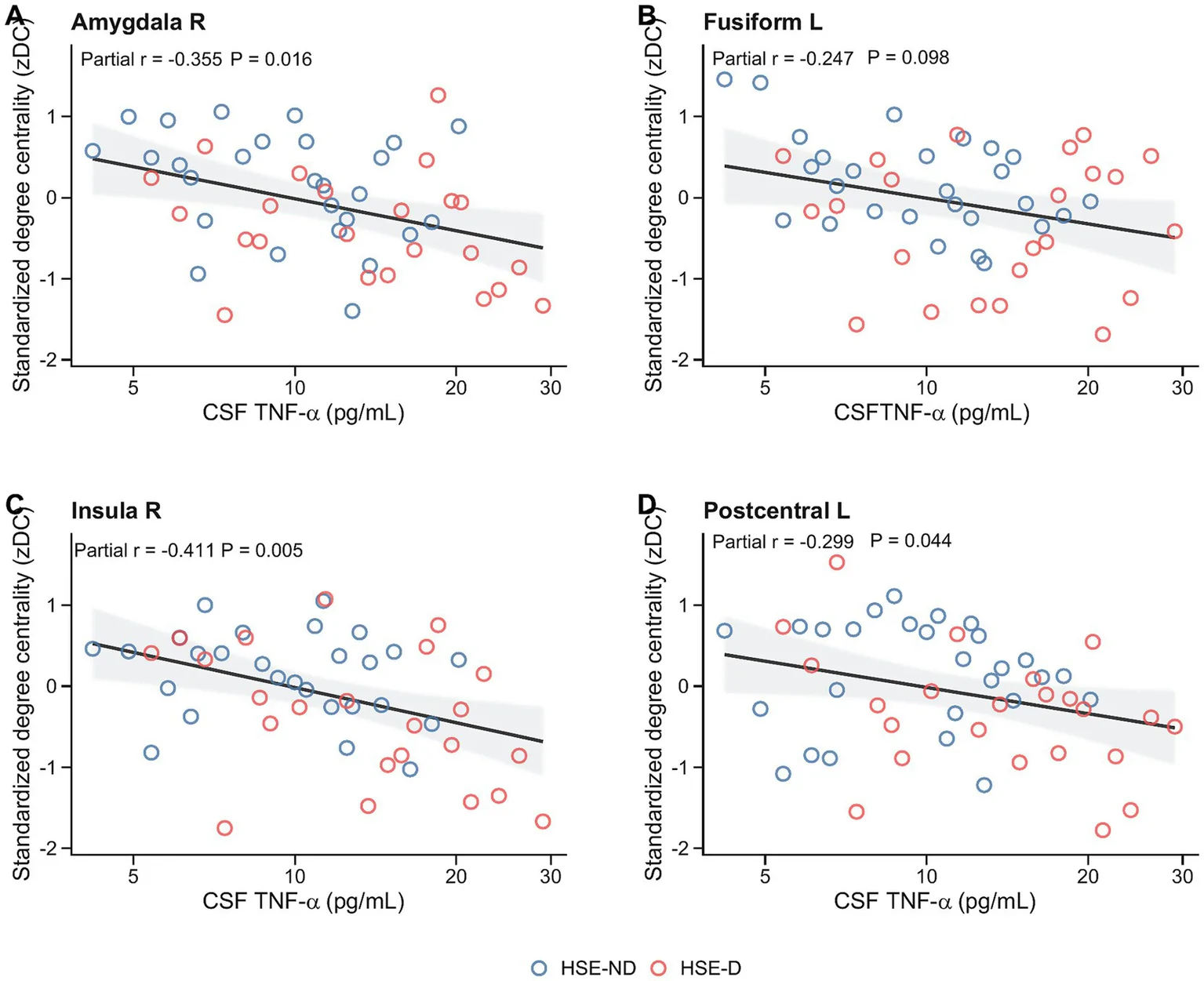
Associations between CSF TNF-α and regional degree centrality. Partial correlation analyses between CSF TNF-α concentrations and regional zDC values in patients with HSE after adjustment for clinical covariates. Associations were evaluated in the right amygdala, left fusiform gyrus, right insula, and left postcentral gyrus.

**Figure 6:**
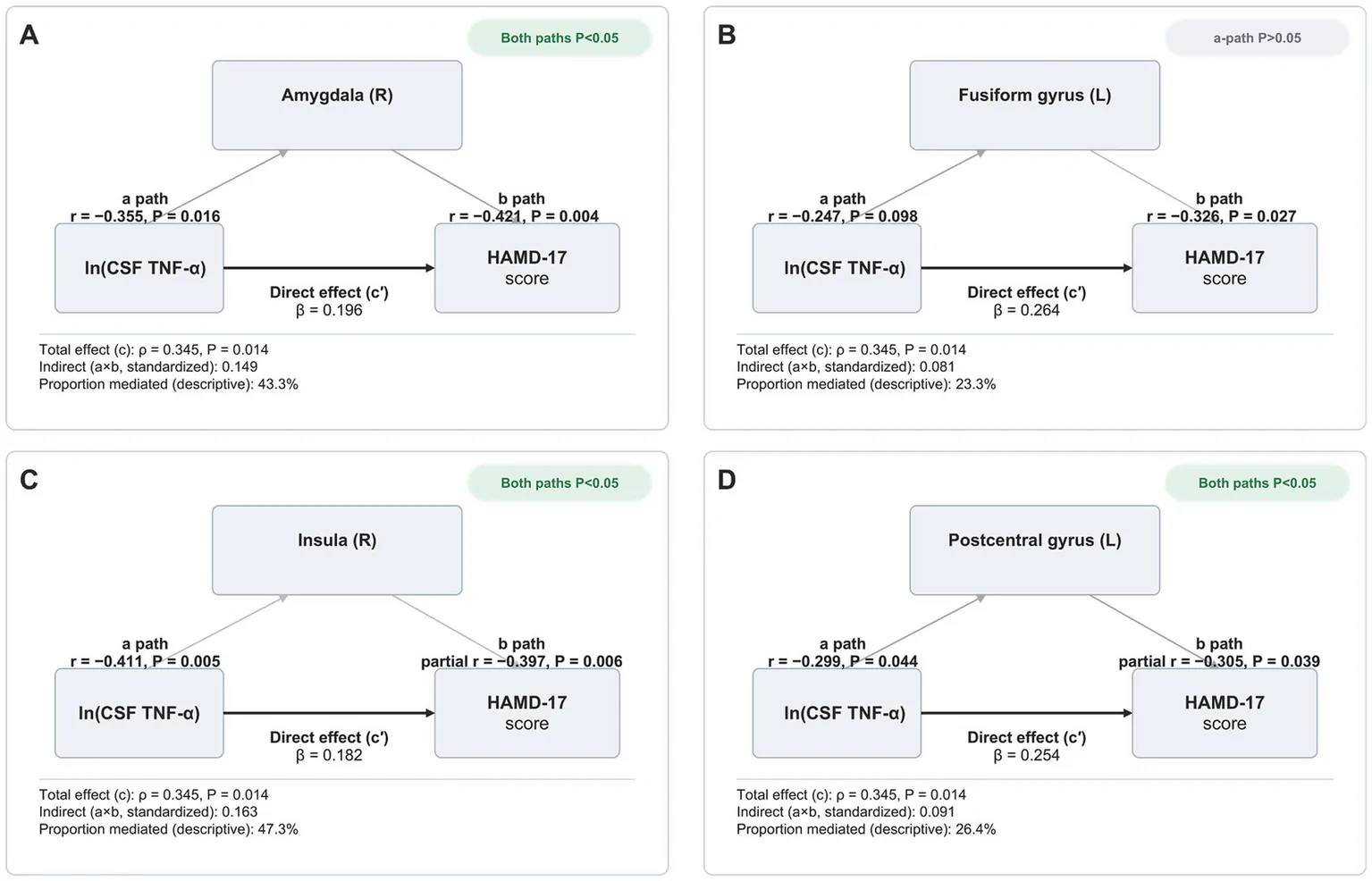
Exploratory path relationships among CSF TNF-α, regional degree centrality, and depressive symptoms. Panels **(A–D)** show the right amygdala, left fusiform gyrus, right insula, and left postcentral gyrus, respectively. The figure displays the a- and b-path association statistics and their *p* values, the direct-effect coefficients (c′), and the standardized indirect-effect point estimates (a × b). Percentages are descriptive. The path-specific *p* values do not constitute tests of the indirect effects. These cross-sectional analyses do not establish causality.

### Multivariable regression analysis

Model 1 had an *R*^2^ of 0.194 (adjusted *R*^2^ = 0.102; F[5, 44] = 2.12; *p* = 0.081). MMSE was inversely associated with HAMD-17 (*B* = −0.54; 95% CI, −1.04 to −0.04; *β* = −0.31; *p* = 0.036). Adding CSF TNF-α in Model 2 increased the in-sample *R*^2^ to 0.302 (adjusted *R*^2^ = 0.205; Δ*R*^2^ = 0.108; F-change *p* = 0.013). CSF TNF-α was positively associated with HAMD-17 in Model 2 (*B* = 2.16; 95% CI, 0.47–3.85; *β* = 0.34; *p* = 0.014). Adding right amygdala and insula zDC in Model 3 increased the in-sample *R*^2^ to 0.421 (adjusted *R*^2^ = 0.308; Δ*R*^2^ = 0.119; F-change *p* = 0.022). Right amygdala zDC remained inversely associated with HAMD-17 (*B* = −1.63; 95% CI, −3.08 to −0.18; *β* = −0.29; *p* = 0.029). Right insula zDC was not significant in Model 3 (*B* = −1.29; 95% CI, −2.68 to 0.10; *β* = −0.23; *p* = 0.069). CSF TNF-α was also not significant in Model 3 (*B* = 1.29; 95% CI, −0.29 to 2.87; *β* = 0.20; *p* = 0.106) (Supplementary Figure S3). Given the sample size, model complexity, and same-sample derivation of the imaging regions, these coefficients and the in-sample *R*^2^ were interpreted as exploratory associations rather than independent or validated predictive effects.

### Exploratory mediation analysis

The standardized indirect-effect estimates were 0.149 for the right amygdala, 0.081 for the left fusiform gyrus, 0.163 for the right insula, and 0.091 for the left postcentral gyrus (Figure 6). These exploratory estimates describe statistical associations and do not establish causal mediation.

## Discussion

We examined CSF cytokines, functional brain organization, and depressive symptoms during recovery from HSE. HSE-D participants showed a broader neuropsychiatric phenotype, with more severe depressive and anxiety symptoms and poorer cognitive performance. CSF TNF-α concentrations were higher in HSE-D and associated with greater depressive symptom severity. CSF IL-6 also differed across groups and correlated with HAMD-17, whereas CSF IL-1β, IL-10, and IL-8 did not. TNF-α was the only CSF cytokine incorporated into the imaging, hierarchical regression, and exploratory mediation analyses. HSE-D also showed lower DC in the right amygdala, right insula, left fusiform gyrus, and left postcentral gyrus. Associations of regional DC with CSF TNF-α and HAMD-17 varied across regions. Adding CSF TNF-α and regional DC sequentially to clinical covariates increased the explained variance in HAMD-17 scores. Exploratory mediation models yielded positive indirect-effect point estimates for all four regions. The findings therefore support a multimodal association framework, not a causal sequence.

HSE outcomes are conventionally judged by acute mortality, seizures, impaired consciousness, and cognitive disability. Neurological stabilization and virological control, however, do not necessarily indicate complete recovery. Persistent affective, behavioral, cognitive, and fatigue-related symptoms are common after encephalitis (1). Depression has received less systematic attention than epilepsy or cognitive impairment, despite its importance for rehabilitation, social reintegration, and quality of life. In our cohort, depressive symptoms coexisted with greater anxiety, lower MMSE and MoCA scores, and a longer index hospitalization. Post-HSE depression therefore appears embedded in a multidimensional recovery phenotype. It should not be interpreted independently of cognition, neurological disability, medication exposure, or acute disease severity. Symptom overlap also complicates classification. Impaired concentration, sleep disturbance, psychomotor slowing, and fatigue can arise from both depressive and neurological syndromes. Structured diagnostic assessment combined with HAMD-17 ratings therefore yielded a more clinically grounded phenotype than a symptom cutoff alone. Follow-up limited to seizures and cognitive screening may underestimate the burden experienced by HSE survivors.

The inflammatory findings extend this clinical pattern. TNF-α participates in innate immune signaling among immune cells, endothelial cells, microglia, and astrocytes. Altered TNF-α signaling has been linked to synaptic regulation, neurovascular integrity, cognitive dysfunction, and depressive symptoms (3). These effects vary with concentration, receptor pathway, timing, and cellular source. CSF TNF-α was prespecified as the primary cytokine for the imaging, hierarchical regression, and exploratory mediation analyses. CSF IL-6 was also higher in HSE-D and correlated with HAMD-17, but it was retained as a secondary cytokine because the prespecified downstream analyses focused on TNF-α. Because all inflammatory biomarkers in this study were measured in CSF, the findings pertain specifically to the central fluid compartment. CSF and peripheral inflammatory concentrations are not interchangeable (16), and no peripheral inflammatory biomarkers were measured in this study. Evidence from a recent systematic review indicates that blood-CSF cytokine relationships are dynamic and context-dependent (17). Paired peripheral and CSF measurements will therefore be needed to evaluate compartmental correspondence after HSE. The CSF TNF-α coefficient was attenuated after regional DC entered the regression model. This attenuation indicates overlapping statistical information; it does not establish that DC changes occurred downstream of TNF-α.

The DC findings complement structural imaging by characterizing distributed functional organization after HSE. Structural MRI is essential for identifying encephalitic injury, and extensive acute abnormalities are associated with poor functional outcome (18). However, focal lesion burden may not capture broader network consequences. Functional abnormalities can extend beyond visible lesions through altered communication among connected regions. DC provides a data-driven measure of each voxel’s connectivity with the rest of the brain. Amygdala-related resting-state connectivity is consistently altered in depression (19). The insula supports salience detection, interoception, and integration of internal and external information (20). The fusiform gyrus supports higher-order visual processing (21), whereas the postcentral gyrus contributes to somatosensory processing and bodily representation. These functions provide context for the regional pattern but do not directly explain depressive symptoms. DC is global and nondirectional. In the secondary ROI analysis, fusiform and insula differences between HSE-D and HSE-ND were not significant after Holm adjustment, so these regions should not be interpreted as depression-specific. Altered DC may reflect depressive symptoms, cognitive status, residual HSE-related injury, anxiety, medication exposure, and other unmeasured factors.

Integrating CSF TNF-α with imaging was the central analytical feature of this study. Previous work has often examined inflammatory biomarkers, structural injury, cognition, and psychiatric outcomes separately. Here, CSF TNF-α was inversely associated with DC in the right amygdala, right insula, and left postcentral gyrus. Regional DC in all four areas was associated with HAMD-17 after adjustment for CSF TNF-α. The HAMA-adjusted sensitivity analysis further examined whether anxiety symptoms accounted for the observed associations between regional DC alterations and depressive symptoms. After adjustment for anxiety severity, the associations remained directionally consistent but were attenuated, indicating that anxiety symptoms may represent an overlapping but incomplete contributor to these relationships. Given the substantial neurobiological overlap between anxiety and depressive symptoms, particularly involving regions such as the amygdala and insula, these findings should not be interpreted as demonstrating depression-specific neural alterations. Rather, they suggest that CSF TNF-α and regional DC changes are associated with depressive symptom severity within the broader neuropsychiatric phenotype following HSE. This pattern does not establish that DC alterations are specific to depressive symptoms. The heterogeneity across regions also does not support a uniform network association with CSF TNF-α. The exploratory indirect-effect point estimates provide hypotheses for longitudinal testing. However, the single-visit design precludes temporal ordering. These cross-sectional analyses may also be underpowered. Regional masks were derived from the same primary contrast, creating potential non-independence in downstream analyses (22). The regression and mediation findings should therefore be interpreted as hypothesis-generating statistical relationships, not as evidence of a biological pathway.

These findings may inform post-HSE care. Neuropsychiatric follow-up should extend beyond seizure surveillance and cognitive testing to include systematic assessment of depression and anxiety (23). This may be especially important after complicated acute courses or when cognitive difficulties persist. Combining clinical assessment with CSF TNF-α and functional imaging may eventually help characterize heterogeneous recovery profiles. However, neither CSF TNF-α nor DC is a validated diagnostic or prognostic biomarker for post-HSE depression. We did not define clinical thresholds, test prospective prediction, or establish utility beyond structured psychiatric assessment. Resting-state fMRI also requires specialized acquisition, processing, and quality control. Clinical translation would require reproducibility across scanners (24), independent validation, calibration, and evidence that testing improves management or outcomes. Longitudinal studies should test whether measurements during acute illness or early recovery predict later neuropsychiatric outcomes. Serial CSF sampling, when clinically and ethically appropriate, could distinguish transient inflammatory changes from persistent phenotypes. Combining repeated sampling with structural and functional imaging may further clarify these trajectories. Whether modifying inflammatory pathways improves neuropsychiatric recovery requires dedicated trials. The present data do not support TNF-targeted treatment.

Study strengths included detailed clinical and neuropsychological characterization, structured depression classification, assessment of multiple CSF cytokines, and integration of CSF TNF-α with resting-state functional MRI. Structural and resting-state MRI were acquired within the same assessment period. Whole-brain DC analysis reduced reliance on a predefined seed, and Gaussian random field correction was applied to the primary imaging contrast. Hierarchical regression evaluated the incremental information from CSF TNF-α and imaging variables rather than testing each domain in isolation. The indirect-effect analyses were explicitly exploratory and included multiple robustness checks. These checks covered alternative bootstrap procedures, clinical and imaging covariates, medication-related analyses, reverse-direction models, and a cognitive negative-control outcome. This approach evaluated the consistency and limitations of the multimodal associations across model specifications.

Several limitations warrant consideration. The cross-sectional observational design precludes conclusions about temporal sequence or causality. The modest sample size limited precision in multivariable and indirect-effect models and restricted analysis of interactions and clinical subgroups. The HSE-D category included heterogeneous DSM-5 depressive-disorder categories rather than a pure primary major-depressive-disorder sample. Longer hospitalization and poorer MMSE/MoCA performance in HSE-D suggest greater neurological burden, and residual confounding by cognitive impairment, lesion location, acute disease severity, medication, sleep disturbance, rehabilitation, vascular risk, psychosocial factors, and shared anxiety and depression symptom variance may remain. The two-center setting, MRI eligibility requirements, and need to complete neuropsychological testing may limit generalizability and introduce survivor or selection bias. Single-time-point CSF cytokine measurements cannot identify cellular source, regional distribution, parenchymal inflammatory activity, or temporal course. The absence of paired peripheral inflammatory measurements prevented evaluation of compartment-specific associations. Complete-case analysis may introduce further bias if data availability was related to illness severity. Model 3 and the mediation analyses were exploratory, and the modest sample size may have produced unstable estimates. DC estimates depend on preprocessing, motion control, gray-matter masking, connectivity threshold, and exclusion of negative correlations. Global-signal handling is one influential preprocessing choice (25). Cluster-based inference can also limit spatial specificity even when the cluster-forming threshold is stringent (26). The reproducibility of resting-state fMRI metrics varies with correction strategy and sample size (27). Finally, correlation and mediation regions were defined in the same sample. Atlas-based regions, split-sample procedures, or independent replication are needed to address potential circularity. Cross-sectional mediation may also misrepresent longitudinal indirect effects (28). The findings should be considered hypothesis-generating.

In this two-center cross-sectional study, CSF TNF-α, regional DC, and depressive symptom severity were statistically associated among HSE survivors. The primary voxel-wise contrast identified candidate DC clusters in four regions, whereas secondary regional comparisons provided stronger HSE-D versus HSE-ND support for the right amygdala and left postcentral gyrus than for the fusiform and insula. The findings do not establish temporal order, cellular source, parenchymal inflammatory activity, depression specificity, or causal relationships. Independent longitudinal studies should use repeated CSF and peripheral measurements and prespecified or externally defined imaging regions to assess reproducibility and clinical relevance.

## Data Availability

The raw data supporting the conclusions of this article will be made available by the authors, without undue reservation.
