# Supplementary material for: Cerebrospinal fluid TNF-α, regional degree centrality, and depressive symptoms after herpes simplex encephalitis: a cross-sectional observational study
Source: Front Neurol. 2026 Oct 16;17:1942369. doi: 10.3389/fneur.2026.1942369 (PMC13725786; doi:10.3389/fneur.2026.1942369)
Supplement: Supplementary file 1 [file Table_1.docx]

**Supplementary Table S1.** MRI acquisition parameters

3.0-T protocol for structural lesion assessment and resting-state degree-centrality analysis

| **Parameter** | **3D T1-weighted anatomical image** | **Axial T2-FLAIR** | **Resting-state BOLD fMRI** |
| --- | --- | --- | --- |
| Scanner | GE Signa HDxt | GE Signa HDxt | GE Signa HDxt |
| Magnetic field strength | 3.0 T | 3.0 T | 3.0 T |
| Receive coil | 8-channel phased-array head coil | 8-channel phased-array head coil | 8-channel phased-array head coil |
| Pulse sequence | 3D inversion-recovery prepared spoiled gradient echo (BRAVO) | 2D fast spin-echo fluid-attenuated inversion recovery | T2*-weighted gradient-echo echo-planar imaging (GE-EPI) |
| Primary purpose | Anatomical registration, segmentation, and gray-matter volume estimation | Chronic lesion/gliosis delineation and lesion-volume covariate | Resting-state functional connectivity and voxel-wise DC |
| Acquisition orientation | Sagittal | Axial, approximately parallel to the AC-PC line | Axial, approximately parallel to the AC-PC line |
| Repetition time (TR) | 8.2 ms | 9,000 ms | 2,000 ms |
| Echo time (TE) | 3.2 ms | 120 ms | 30 ms |
| Inversion time (TI) | 450 ms | 2,500 ms | Not applicable |
| Flip angle | 12° | 160° refocusing angle | 90° |
| Field of view | 256 × 256 mm² | 240 × 240 mm² | 240 × 240 mm² |
| Acquisition matrix | 256 × 256 | 256 × 256 | 64 × 64 |
| Number of slices | 188 contiguous slices | 36 slices | 37 slices |
| Slice thickness / gap | 1.0 mm / 0 mm | 4.0 mm / 0.5 mm | 4.0 mm / 0 mm |
| Acquired voxel size | 1.0 × 1.0 × 1.0 mm³ | 0.94 × 0.94 × 4.0 mm³ | 3.75 × 3.75 × 4.0 mm³ |
| Number of volumes | Not applicable | Not applicable | 210 volumes |
| Slice acquisition order | 3D acquisition | Interleaved | Interleaved ascending |
| Phase-encoding direction | Anterior-to-posterior | Anterior-to-posterior | Anterior-to-posterior |
| Parallel imaging | ASSET factor 2 | None | None |
| Nominal acquisition time | 4 min 48 s | 4 min 30 s | 7 min 00 s |
| Participant instructions | Remain still; foam padding used for head stabilization | Remain still; foam padding used for head stabilization | Eyes closed, remain awake, and relax without structured thinking; wakefulness confirmed immediately after scanning |
| Use in analysis | Unified segmentation and normalization to MNI space | Manual or semi-automatic lesion mask reviewed by a neuroradiologist | First 10 volumes discarded; remaining 200 volumes entered into preprocessing |

**Supplementary Table S2.** Medication and clinical treatment information

Acute-phase treatment and medication exposure at MRI assessment in HSE patients

| **Treatment variable** | **HSE-D (n = 23)** | **HSE-ND (n = 27)** | **P value** | **Clinical/statistical note** |
| --- | --- | --- | --- | --- |
| **A. Acute-phase HSE treatment** | | | | |
| Intravenous acyclovir, n (%) | 23 (100.0) | 27 (100.0) | - | All participants received first-line antiviral therapy. |
| Antiviral initiated within 24 h of admission, n (%) | 19 (82.6) | 24 (88.9) | 0.689 | Small between-group imbalance without evidence of delayed-treatment confounding. |
| Acyclovir treatment duration, days | 19 (17-20.5) | 18 (16.5-20) | 0.392 | Values are median (IQR); all courses were within 14-21 days. |
| Renal dose adjustment during acyclovir, n (%) | 4 (17.4) | 3 (11.1) | 0.689 | Adjustment reflected transient renal impairment or reduced creatinine clearance. |
| Adjunctive acute corticosteroid treatment, n (%) | 9 (39.1) | 8 (29.6) | 0.557 | Consistent with the corresponding treatment totals reported in Table 1. |
| Methylprednisolone-based regimen, n (%) | 6 (26.1) | 5 (18.5) | 0.733 | Nested within corticosteroid-treated participants. |
| Dexamethasone-based regimen, n (%) | 3 (13.0) | 3 (11.1) | 1.000 | Nested within corticosteroid-treated participants. |
| Corticosteroid duration among treated patients, days | 5 (3-7) | 4.5 (3-6) | 0.655 | Short adjunctive courses; no participant remained on corticosteroids at MRI. |
| Acute antiseizure medication, n (%) | 14 (60.9) | 13 (48.1) | 0.407 | Treatment of acute symptomatic seizures or epileptiform activity. |
| Intensive care unit admission, n (%) | 8 (34.8) | 6 (22.2) | 0.361 | Slightly greater acute severity in HSE-D, without a statistically significant difference. |
| Mechanical ventilation, n (%) | 4 (17.4) | 3 (11.1) | 0.689 | Supportive treatment during the acute encephalitic episode. |
| **B. Medication and rehabilitation status at MRI assessment** | | | | |
| Current antiseizure medication, n (%) | 8 (34.8) | 7 (25.9) | 0.548 | Stable regimen; no dose change within 2 weeks before MRI. |
| **Current antidepressant treatment, n (%)** | **6 (26.1)** | **1 (3.7)** | **0.039** | **More common in HSE-D; adjust for this exposure or evaluate it in sensitivity analyses.** |
| Selective serotonin reuptake inhibitor, n (%) | 4 (17.4) | 1 (3.7) | 0.167 | Examples include sertraline or escitalopram; nested within antidepressant use. |
| Serotonin-norepinephrine reuptake inhibitor, n (%) | 2 (8.7) | 0 (0.0) | 0.207 | Examples include venlafaxine or duloxetine; nested within antidepressant use. |
| Benzodiazepine or non-benzodiazepine hypnotic, n (%) | 4 (17.4) | 2 (7.4) | 0.395 | PRN sedatives were withheld for at least 24 h before MRI when clinically feasible. |
| Antipsychotic medication, n (%) | 1 (4.3) | 0 (0.0) | 0.460 | Low-frequency exposure; no participant received a high-dose regimen. |
| Current systemic corticosteroid or other immunomodulator, n (%) | 0 (0.0) | 0 (0.0) | - | Reduces direct treatment-related confounding of inflammatory biomarker levels. |
| Structured rehabilitation after discharge, n (%) | 14 (60.9) | 14 (51.9) | 0.577 | Included cognitive, physical, occupational, or speech rehabilitation. |

**Supplementary Table S3. CSF cytokine concentrations among groups**

| **CSF cytokine** | **HSE-D (n = 23)** | **HSE-ND (n = 27)** | **HC (n = 24)** | **P value** | **Post hoc comparison** |
| --- | --- | --- | --- | --- | --- |
| TNF-α, pg/mL | 14.9 (8.8–20.0) | 10.5 (6.7–13.0) | 7.8 (6.4–10.4) | 0.012 | HSE-D > HC (0.010); HSE-D > HSE-ND (0.043) |
| IL-6, pg/mL | 4.9 (3.0–7.4) | 3.6 (2.2–5.3) | 2.4 (1.5–3.8) | 0.021 | HSE-D > HC (0.018) |
| IL-1β, pg/mL | 1.14 (0.66–1.86) | 0.97 (0.56–1.52) | 0.81 (0.47–1.26) | 0.184 | No significant pairwise difference |
| IL-10, pg/mL | 2.8 (1.7–4.4) | 2.4 (1.5–3.7) | 1.8 (1.1–2.7) | 0.076 | Not performed (overall P > 0.05) |
| IL-8, pg/mL | 7.3 (4.6–10.9) | 6.1 (3.9–9.2) | 4.8 (3.0–7.1) | 0.068 | Not performed (overall P > 0.05) |

**Supplementary Table S4.** Brain regions showing DC differences before and after GRF correction

Primary whole-brain contrast: HSE-D versus HSE-ND

| **Brain region** | **Hemisphere** | **Cluster size (voxels)** | **MNI coordinates (x, y, z)** | **Peak T-score** | **Cluster-level GRF P** |
| --- | --- | --- | --- | --- | --- |
| **A. Candidate clusters before GRF correction** | | | | | |
| Amygdala | R | 91 | 24, -3, -18 | -4.21 | **0.018** |
| Insula | R | 86 | 36, 15, 0 | -4.05 | **0.024** |
| Fusiform gyrus | L | 73 | -39, -48, -18 | -3.86 | **0.031** |
| Postcentral gyrus | L | 69 | -42, -24, 51 | -3.68 | **0.044** |
| Hippocampus | L | 41 | -27, -18, -15 | -3.60 | 0.076 |
| Parahippocampal gyrus | R | 36 | 24, -30, -12 | -3.52 | 0.091 |
| Superior temporal gyrus | L | 32 | -51, -18, 6 | -3.44 | 0.118 |
| Precuneus | R | 29 | 9, -57, 45 | -3.37 | 0.147 |
| Medial superior frontal gyrus | L | 27 | -6, 48, 33 | -3.29 | 0.183 |
| **B. Clusters surviving cluster-level GRF correction** | | | | | |
| **Amygdala** | **R** | **91** | **24, -3, -18** | **-4.21** | **0.018** |
| **Insula** | **R** | **86** | **36, 15, 0** | **-4.05** | **0.024** |
| **Fusiform gyrus** | **L** | **73** | **-39, -48, -18** | **-3.86** | **0.031** |
| **Postcentral gyrus** | **L** | **69** | **-42, -24, 51** | **-3.68** | **0.044** |

**Supplementary Table S5.** Mean standardized DC values and statistical comparisons among groups

Secondary characterization of the four GRF-surviving brain regions

| **Brain region** | **HC (n = 24)** | **HSE-ND (n = 27)** | **HSE-D (n = 23)** | **F (2, 71)** | **Overall P** | **HSE-D vs HC Holm P** | **HSE-D vs HSE-ND Holm P** | **HSE-ND vs HC Holm P** |
| --- | --- | --- | --- | --- | --- | --- | --- | --- |
| Right amygdala | 0.27 ± 0.82 | 0.08 ± 0.85 | −0.49 ± 0.83 | 5.28 | **0.007** | **0.009** | **0.042** | 0.422 |
| Left fusiform gyrus | 0.22 ± 0.78 | 0.03 ± 0.80 | −0.42 ± 0.79 | 4.06 | **0.021** | **0.023** | 0.104 | 0.396 |
| Right insula | 0.25 ± 0.78 | 0.08 ± 0.83 | −0.39 ± 0.82 | 3.92 | **0.024** | **0.026** | 0.101 | 0.456 |
| Left postcentral gyrus | 0.28 ± 0.82 | 0.06 ± 0.85 | −0.50 ± 0.83 | 5.46 | **0.006** | **0.007** | **0.046** | 0.353 |

**Supplementary Table S6.** Correlations between CSF cytokines and clinical variables

Exploratory associations in the pooled HSE imaging cohort (n = 50)

| **CSF cytokine** | **HAMD-17 score ρ (P value)** | **MMSE score ρ (P value)** | **Disease duration ρ (P value)** | **Hospitalization duration ρ (P value)** |
| --- | --- | --- | --- | --- |
| **TNF-α, pg/mL** | **0.345 (0.014)** | **−0.184 (0.200)** | **0.072 (0.621)** | **0.120 (0.406)** |
| IL-6, pg/mL | **0.288 (0.042)** | −0.210 (0.143) | 0.174 (0.228) | 0.189 (0.189) |
| IL-1β, pg/mL | 0.169 (0.242) | −0.163 (0.258) | 0.064 (0.657) | 0.140 (0.331) |
| IL-10, pg/mL | 0.114 (0.429) | 0.071 (0.624) | −0.022 (0.880) | 0.149 (0.303) |
| IL-8, pg/mL | 0.151 (0.296) | −0.139 (0.337) | 0.097 (0.504) | 0.102 (0.483) |
